# Outcomes of Vitrectomy Combined with Scleral Buckling for Eyes with Early Recurrence of Simple Rhegmatogenous Retinal Detachment Previously Treated by Pars Plana Vitrectomy

**DOI:** 10.1155/2020/6637143

**Published:** 2020-12-17

**Authors:** Tomoaki Tatsumi, Takayuki Baba, Takehito Iwase, Tomohiro Nizawa, Gen Miura, Hirotaka Yokouchi, Shuichi Yamamoto

**Affiliations:** Department of Ophthalmology and Visual Science, Chiba University Graduate School of Medicine, Chiba, Japan

## Abstract

**Purpose:**

To investigate the outcomes of pars plana vitrectomy (PPV) combined with scleral buckling (SB) in treating eyes with an early recurrent rhegmatogenous retinal detachment (rRRD).

**Methods:**

This was a retrospective, interventional case series of 21 eyes with an early rRRD treated by PPV combined with SB. The significance of the associations between the variants before the surgeries and the final best-corrected visual acuity (BCVA) was determined.

**Results:**

The average age of the patients was 61.0 ± 9.6 years. A retinal reattachment was observed in 20 of 21 eyes (95.2%) after a single reoperation. The BCVA was 0.91 ± 0.90 logMAR units before the initial surgery and 0.94 ± 0.94 logMAR units before the reoperations, and it improved significantly to 0.49 ± 0.50 logMAR units after the reoperation (*P* = 0.016, *P* = 0.002, respectively). The preoperative BCVA was significantly correlated with the final BCVA (*P* = 0.043, *r* = 0.445 before the primary surgery; *P* < 0.001, *r* = 0.885 before reoperation).

**Conclusions:**

The reattachment of an early recurrent retinal detachment by PPV with SB is effective.

## 1. Introduction

A rhegmatogenous retinal detachment (RRD) is a vision-threatening disorder, but recent advances of surgical techniques and instruments have improved the anatomical and functional outcomes. The average success rate for RRD is over 90% after a single reattachment surgery and almost 100% after multiple surgeries [1–3]. A significant trend has been reported toward the use of pars plana vitrectomy (PPV) as the primary surgery for RRD eyes with a detachment of the posterior hyaloid membrane [4].

Despite the increased rate of success after the primary surgery, the retina of some eyes can detach again. Nevertheless, the outcomes after surgeries for a recurrent RRD (rRRD) have been rarely reported [5–8]. A large case series including the various baseline characteristics, i.e., proliferative vitreoretinopathy (PVR), giant tear, and the presence of a macular hole, has been reported [6, 7]. The various types of primary interventions include PPV, scleral buckling (SB), and combination of both, and various secondary attempts include repeat PPV alone, buckle revision, combination of PPV and SB, and intraocular tamponade with silicone oil (SO) or expanding gas. The results of these studies have been helpful for understanding the overall success rate, but they are less helpful in terms of making a decision of the type of treatment in an individual case.

The time of the redetachment of the initially reattached retina can be designated as an early and a late recurrence [9]. The time of the recurrence is important because the incidence and cause for the detachment varies, and earlier studies did not take the time of the redetachment into consideration for the final outcomes.

Therefore, the purpose of this study was to determine the anatomical and functional outcomes after PPV combined with SB for repairing an early rRRD which had been treated by PPV alone. We also investigated the factors that were significantly correlated with the final visual acuity.

## 2. Patients and Methods

This was a nonrandomized review of the medical charts of 21 eyes of 21 patients that had an early redetachment after a successful reattachment of an RRD. All cases were treated at the Chiba University Hospital between October 2012 and October 2019. During this period, 1201 eyes had undergone PPVs for repairing an RRD. Among these cases, the eyes with a redetachment of the RRD within 2 months after the successful initial PPV for the treatment of a RRD were studied [9]. The eyes with a redetachment within 14 days after the primary surgery were excluded because those cases were classified as a primary failure. Eyes with vision-affecting diseases such as glaucoma, corneal disorders, retinal vascular disorders including diabetic retinopathy, and dense cataracts were excluded. The RRDs due to giant tear, macular hole, and RRD secondary to ocular trauma were also excluded. The eyes with PVR grade C or above were excluded [10].

The procedures used in this study were approved by the Institutional Review Board of Chiba University Graduate School of Medicine (No. 3746), and they conformed to the tenets of the Declaration of Helsinki. The procedures to be used and the purpose of this study were provided to all patients, and a signed informed consent was obtained from all. The consent form also included permission to use the information collected for future research and publications with preservation of the anonymity.

The data collected from the medical charts were the age, sex, laterality, best-corrected visual acuity (BCVA), time from onset of the RRD to the initial surgery, refractive error (spherical equivalent), axial length (AL), intraocular pressure (IOP), PVR grade A or B, presence of macular detachment, choroidal detachment, vitreous hemorrhage, status of crystalline lens, number of retinal breaks, location of the largest retinal break, number of quadrants of detached retina, use of concomitant phacoemulsification, number of photocoagulations, material used for intraocular tamponade, intraoperative use of triamcinolone, perfluorocarbon liquid, removal of internal limiting membrane, and the incidence of complications. These factors collected before the primary PPVs and before the reoperations were analyzed. In addition, the time from the initial surgery to the redetachment of the RRD and the cause of the redetachment was recorded.

The BCVA was measured with a Snellen chart, and the decimal BCVA was converted to the logarithm of minimal angle of resolution (logMAR) units for the statistical analyses. The lower visual acuities of hand motion = 3.00 logMAR units and counting fingers = 2.00 logMAR units as reported [11, 12]. The BCVA at the last visit was used as the final BCVA.

### 2.1. Pars Plana Vitrectomy

The surgeries were performed by two experienced vitreoretinal surgeons (TB, TT). The surgical procedures consisted of standard pars plana vitrectomy with 25-gauge instruments. First, a conjunctival peritomy was performed, and mattress sutures were placed for fixing the buckle. In some cases, scleral tunnels were used instead of mattress sutures. After removing the residual vitreous gel, the subretinal fluid was aspirated, endolaser coagulation was applied around the retinal breaks after fluid-air exchange, the encircling or segmental buckle was fixed, and endotamponade with sulfur hexafluoride gas (SF6) or silicone oil (SO) was performed. The use of SF6 or SO was determined by the surgeon's preference. A large part of the subretinal fluid was removed through the existing break with fluid-air exchange. A small amount of residual fluid at the posterior pole was present at the end of surgery. The use of segmental or encircling buckle was at the surgeon's discretion. A silicone sponge of 5 mm width or silicone tire of 7 mm width was used as buckling elements. A 5 mm-wide silicone sponge was used for the segmental buckle, and a 7 mm-wide silicone tire was used for the encircling surgery. Relaxing retinotomy was not performed in any case. We advised the patients to maintain a face-down position for 7 to 10 days for those with a SF6 tamponade and one day for the patients with SO tamponade.

### 2.2. Statistical Analyses

The significance of the changes in the BCVA was determined by Wilcoxon's rank sum test. The significance of the differences in the values of the two groups was determined by the Mann–Whitney tests, and that for three groups or more were determined by the Kruskal–Wallis tests. The significance of the correlations between the final BCVA and before the primary PPV and that before the reoperation were determined by Spearman's rank correlation coefficient test. A *P* value <0.05 was taken to be statistically significant. The uneventful removal of SO was not counted as additional reoperation or failure of surgery.

## 3. Results

The average ± SD of the age of the cases was 61.0 ± 9.6 years. There were 8 women (38%), and 12 of the 21 eyes (57%) had an RRD in the right eye. The mean interval between the initial surgery and the redetachment was 25.1 ± 11.3 days. The postoperative observation period after the second surgery was 10.5 ± 7.2 months with a range of 3 to 30 months.

**Figure 1:**
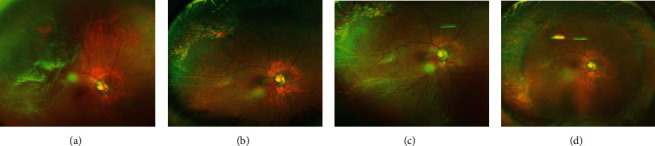
Findings in a 71-year-old Japanese woman with a recurrent rhegmatogenous retinal detachment after successful attachment by pars plana vitrectomy. She was treated by a second surgery with vitrectomy and encircling scleral buckling. (a) Ultra-wide-field fundus image showing a retinal break at the superotemporal area and rhegmatogenous retinal detachment (RRD). Her best-corrected visual acuity (BCVA) was 20/30. (b) Ultra-wide-field fundus image after the initial surgery showing retinal scar and attached retina. (c) The recurrence of RRD was observed at 2 weeks after the initial surgery. Ultra-wide-field fundus image shows inferior retinal detachment. The cause of the RRD was a new small break at the inferonasal retina. Her BCVA was 20/60. (d): Ultra-wide-field fundus image after a reoperation by vitrectomy and encircling buckle. Reattached retina and the protrusion of encircling buckle can be seen. Her visual acuity was 20/25 at seven months after the reoperation.

A representative case is presented in Figure 1.

A retinal reattachment was achieved in 20/21 eyes (95.2%) after a single surgery. SO was used in 9 eyes and successfully removed in all eyes within 3 to 7 months. A reattachment was observed in all of eyes at the final visit.

**Figure 2:**
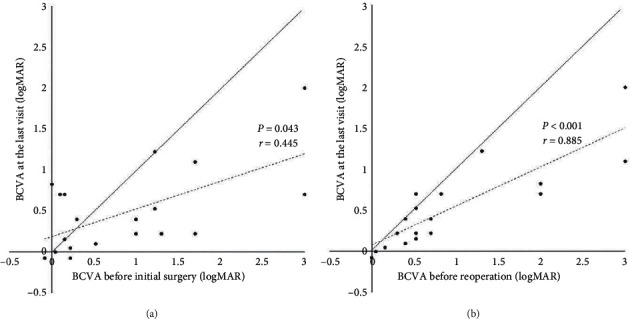
Visual acuity before the initial surgery, before the surgery for the recurrence, and at the last visit. (a) Best-corrected visual acuity (BCVA) before the initial surgeries and at the last visit. A better final BCVA was associated with the better BCVA before the initial surgeries (*r* = 0.445, *P* = 0.043). (b) BCVA before the surgeries for the recurrent detachment and at the last visit. A better last BCVA was associated with the better BCVA before the surgery for the recurrent retinal detachment (*r* = 0.885, *P* < 0.001).

The BCVA was 0.91 ± 0.90 logMAR units before the initial surgery, 0.94 ± 0.94 logMAR units before the reoperations, and 0.49 ± 0.50 logMAR units at the final visit (Figure 2). The final BCVA was significantly better than that at both earlier times (*P* = 0.016, *P* = 0.002, respectively). Significant and positive correlations were found between the final BCVA and the BCVA before the initial surgery and before the reoperation (*r* = 0.445, *P* = 0.043; *r* = 0.885, *P* < 0.001, respectively).

The associations between the baseline characteristics and the final BCVA are summarized in Table 1. The presence of macular-off retinal detachment and choroidal detachment at the baseline was negatively associated with a final BCVA (*P* = 0.018, *P* = 0.010, respectively).

The associations between the values of the different factors before the reoperation and the final visual acuity are summarized in Table 2. The presence of a choroidal detachment was significantly and negatively associated with the final BCVA (*P* = 0.010). A detachment of the macula before the reoperation was associated with a poorer BCVA at the final examination, but the difference was not significant (*P* = 0.051).

The correlations between the final BCVA and the age (*P* = 0.350), interval between the time of onset to surgery (*P* = 0.557), interval between initial surgery and reoperation (*P* = 0.664), IOP (*P* = 0.302 before initial surgery; *P* = 0.376 before second surgery), axial length (*P* = 0.792), refractive error (*P* = 0.366), and number of endophotocoagulations (*P* = 0.329 at initial surgeries, *P* = 0.462 at reoperations) were not significant.

Iatrogenic retinal breaks during the initial PPV were found in two eyes. One eye developed a PVR after the reoperation and required three PPVs. The retina was eventually attached, but the vision was limited because of severe bullous keratopathy. Two eyes had an increase in the IOP which was controlled by topical glaucoma medications.

## 4. Discussion

Our results showed that a retinal reattachment was achieved in 95.2% of eyes with rRRD by a single PPV combined with SB. The BCVA at the final visit was positively and significantly associated with the BCVA before the primary PPVs and with the BCVA before the reoperation. The presence of a macular detachment and choroidal detachment before the primary surgery and reoperation was significantly correlated with a poorer final BCVA.

We suggest that the redetachments were caused due to the development of new breaks, undetected breaks at the initial surgery, a reopening of treated breaks, or PVR [13]. The reopening of breaks suggests that a traction by residual vitreous cortex may have induced the rRRD. Surgeons can reduce the traction by SB. There were also new breaks, and we assume that the residual vitreous may have created traction to induce these other breaks. Therefore, the reduction of traction by residual vitreous strands by SB was essential. This is especially true if we did not use SB during the initial PPV.

Another option to counteract the peripheral vitreous traction is a 360-degree laser retinopexy, but recent data suggest that there is no benefit of this in increasing the reattachment rate [14].

The mean interval between the initial surgery and the redetachment was 25.1 ± 11.3 days. We focused on the early recurrence of retinal detachment by excluding cases with a redetachment that developed more than two months after the primary surgery. By limiting cases to those that had recurrence within two months, we assume that the cause of redetachment was an early contraction of the peripheral vitreous because the proliferative tissue did not form in such a short time. We suggest that the additional support of the peripheral retina by the scleral buckle is enough for simple recurrence due to the early contraction of the residual vitreous.

The results of recent studies have also shown that the primary success rate after rRRD is between 65 and 83% [5–8]. In our cohort, the primary success rate was 95.2%. The previous studies with large number of rRRD cases included a variety of cases, e.g., giant tear, macular hole, and advanced PVR, which had been treated by various surgeries, e.g., SB, PPV, and PPV + SB, which might have relatively low 66.2 to 68.3% reattachment rates [6, 7]. We suggest that our results were better because we studied relatively uncomplicated rRRD cases.

The use of silicone oil tamponade has been reported to be effective in the overall success rate of reattachment of complicated RRD cases with PVR [15]. We did not find any advantages of using a silicone oil tamponade in our cases probably because none had severe PVR, and the intraocular inflammation was relatively mild. We suggest that the SF6 gas is sufficient in achieving favorable results in treating simple rRRD eyes.

There was a trend towards a poorer final visual acuity in eyes that used PFCL than in eyes without the use of PFCL. The number of quadrants detached was greater in eyes with PFCL than in eyes without PFCL use (2.8 ± 1.3 vs 2.4 ± 1.0) which might have affected the final visual acuity. We occasionally used PFCL to detect small peripheral breaks, in which case the use of PFCL was not related to the final visual acuity.

We did not perform retinotomy in any of our cases even though retinotomy has been reported to facilitate reattachment in eyes with a RRD [16]. However, the recurrence of PVR and persistent hypotony are the adverse effects of this technique. The other option for treating complicated RRDs is scleral buckling which reduces the traction on the peripheral retina by residual peripheral vitreous. This less invasive technique should be considered before using a large-area retinotomy.

This study has limitations. First, the number of cases was relatively small. We included only the early rRRD cases after primary PPV to determine the efficacy of PPV + SB for simple vitrectomized rRRD cases. We also excluded the complicated rRRD eyes such as those with severe PVR, giant tear, and macular hole, which may require a different treatment strategy. Second, there was no control for the PPV + SB. We should compare the result after repeated PPV alone to PPV + SB, but we usually select PPV + SB for the rRRDs at our institution because of practical and socioeconomic reasons to avoid any further costly interventions. A prospective study to compare PPV and PPV + SB for rRRDs is necessary although the number of rRRD is getting smaller.

In conclusion, we found that the PPV combined with SB was effective in treating early, simple rRRD, and the anatomical success rate was very high. This technique should be considered before any other aggressive maneuvers such as 360-degree laser retinopexy or wide retinotomy are used.

## Figures and Tables

**Table 1:** Relationship between factors before initial surgery and the final visual acuity.

	BCVA (logMAR)			*P* value
	*N* (eyes)	Average ± SD	95% CI	
Sex				0.414^*∗*^
Men	13 (62%)	0.59 ± 0.58	0.23, 0.94	
Women	8 (38%)	0.33 ± 0.29	0.09, 0.58	
Laterality				0.651^*∗*^
Right	12 (57%)	0.56 ± 0.59	0.18, 0.93	
Left	9 (43%)	0.40 ± 0.36	0.12, 0.68	
Lens status				0.197^*∗*^
Phakia	14 (67%)	0.36 ± 0.35	0.16, 0.56	
Pseudophakia	7 (33%)	0.75 ± 0.68	0.12, 1.38	
Macular detachment				0.018^*∗*^
Present	17 (81%)	0.59 ± 0.50	0.33, 0.85	
Absent	4 (19%)	0.07 ± 0.23	−0.29, 0.43	
Quadrants of detachment				0.193^*∗∗*^
1	3 (14%)	0.15 ± 0.22	−0.39, 0.69	
2	10 (48%)	0.39 ± 0.40	0.10, 0.67	
3	2 (10%)	0.46 ± 0.34	−2.57, 3.49	
4	6 (29%)	0.84 ± 0.67	0.15, 1.53	
Choroidal detachment				0.010^*∗*^
Present	2 (10%)	1.61 ± 0.55	−3.33, 6.55	
Absent	19 (90%)	0.37 ± 0.33	0.21, 0.53	
Vitreous hemorrhage				0.887^*∗*^
Present	3 (14%)	0.47 ± 0.59	−0.99, 1.94	
Absent	18 (86%)	0.49 ± 0.50	0.24, 0.74	
Number of retinal breaks				0.223^*∗∗*^
1	8 (38%)	0.60 ± 0.38	0.28, 0.92	
2	0 (0%)	NA	NA	
3	8 (38%)	0.24 ± 0.36	−0.06, 0.54	
4	3 (14%)	0.46 ± 0.28	−0.23, 1.15	
5-	2 (10%)	1.11 ± 1.26	−10.18, 12.41	
Location of the largest break				0.699^*∗∗*^
Superotemporal	17 (81%)	0.50 ± 0.51	0.24, 0.76	
Inferotemporal	2 (10%)	0.66 ± 0.80	−6.49, 7.81	
Superonasal	1 (5%)	0.05	NA	
Inferonasal	1 (5%)	0.4	NA	
Proliferative viteroretinopathy				0.842^*∗*^
Grade A	16 (76%)	0.49 ± 0.53	0.20, 0.77	
Grade B	5 (24%)	0.49 ± 0.44	−0.05, 1.03	
Concurrent cataract surgery				0.197^*∗*^
Present	14 (67%)	0.36 ± 0.35	0.16, 0.56	
Absent	7 (33%)	0.75 ± 0.68	0.12, 1.38	
Intraocular tamponade				0.221^*∗*^
Room air	3 (14%)	0.21 ± 0.43	−0.86, 1.27	
SF6	18 (86%)	0.54 ± 0.51	0.28, 0.79	
Removal of ILM				1.000^*∗*^
Absent	21 (100%)	0.49 ± 0.50	0.26, 0.72	
Use of triamcinolone				0.762^*∗*^
Present	20 (95%)	0.50 ± 0.51	0.26, 0.74	
Absent	1 (5%)	0.22	NA	
Use of perfluorocarbon liquid				0.267^*∗*^
Present	6 (29%)	0.63 ± 0.47	0.14, 1.12	
Absent	15 (71%)	0.43 ± 0.52	0.15, 0.72	
Iatrogenic retinal breaks				0.467^*∗*^
Present	2 (10%)	0.22 ± 0.25	−2.01, 2.45	
Absent	19 (90%)	0.52 ± 0.52	0.27, 0.77	

BCVA: best-corrected visual acuity. logMAR: logarithm of minimal angle of resolution. SD: standard deviation. CI: confidence interval. NA: not applicable. SF6: sulfur hexafluoride. ILM: internal limiting membrane. ^*∗*^Mann–Whitney test. ^*∗∗*^Kruskal–Wallis test.

**Table 2:** Relationship between factors before surgery for recurrent retinal detachment and the final visual acuity.

	BCVA (logMAR)			*P* value
	*N* (eyes)	Average ± SD	95% CI	
Reason for recurrence of RRD				0.091^*∗*^
Reopen of primary breaks	16 (76%)	0.59 ± 0.53	0.30, 0.87	
New retinal breaks	5 (24%)	0.19 ± 0.20	−0.07, 0.45	
Macular detachment				0.051^*∗*^
Present	11 (52%)	0.70 ± 0.59	0.31, 1.10	
Absent	10 (48%)	0.26 ± 0.25	0.08, 0.43	
Quadrants of detachment				0.146^*∗∗*^
1	2 (10%)	0.37 ± 0.46	−3.78, 4.52	
2	8 (38%)	0.21 ± 0.19	0.05, 0.37	
3	4 (19%)	0.50 ± 0.33	−0.01, 1.02	
4	7 (33%)	0.84 ± 0.67	0.21, 1.46	
Choroidal detachment				0.010^*∗*^
Present	2 (10%)	1.61 ± 0.55	−3.33, 6.55	
Absent	19 (90%)	0.37 ± 0.33	0.21, 0.53	
Vitreous hemorrhage				1.000^*∗*^
Absent	21 (100%)	0.49 ± 0.50	0.26, 0.72	
Number of retinal breaks				0.269^*∗∗*^
1	13 (62%)	0.52 ± 0.37	0.30, 0.75	
2	3 (14%)	0.08 ± 0.28	−0.60, 0.76	
3	0 (0%)	NA	NA	
4	2 (10%)	0.38 ± 0.46	−3.77, 4.52	
5-	3 (14%)	0.83 ± 1.02	−1.71, 3.37	
Location of the largest break				0.366^*∗∗*^
Superotemporal	13 (62%)	0.55 ± 0.60	0.19, 0.91	
Inferotemporal	2 (10%)	0.76 ± 0.09	−0.03, 1.56	
Superonasal	1 (5%)	0.4	NA	
Inferonasal	5 (24%)	0.23 ± 0.18	0.01, 0.45	
Proliferative viteroretinopathy				1.000^*∗*^
Grade B	21 (100%)	0.49 ± 0.50	0.26, 0.72	
Concurrent cataract surgery				1.000^*∗*^
Absent	21 (100%)	0.49 ± 0.50	0.26, 0.72	
Location of scleral buckle				0.887^*∗*^
Encircling	18 (86%)	0.50 ± 0.54	0.23, 0.77	
Segmental	3 (14%)	0.44 ± 0.24	−0.16, 1.04	
Intraocular tamponade				0.883^*∗*^
SF6	12 (57%)	0.55 ± 0.62	0.13, 0.97	
Silicone oil	9 (43%)	0.44 ± 0.36	0.17, 0.72	
Removal of ILM				0.534^*∗*^
Present	3 (14%)	0.56 ± 0.36	−0.32, 1.44	
Absent	18 (86%)	0.48 ± 0.53	0.22, 0.74	
Use of triamcinolone				0.771^*∗*^
Present	19 (90%)	0.51 ± 0.52	0.26, 0.76	
Absent	2 (10%)	0.31 ± 0.12	−0.81, 1.43	
Use of perfluorocarbon liquid				1.000^*∗*^
Present	13 (62%)	0.52 ± 0.59	0.16, 0.88	
Absent	8 (38%)	0.44 ± 0.34	0.16, 0.73	
Postoperative complications				0.093^*∗*^
Present	1 (5%)	1.85	NA	
Absent	20 (95%)	0.41 ± 0.37	0.21, 0.54	

BCVA: best-corrected visual acuity. LogMAR: logarithm of minimal angle of resolution. SD: standard deviation. CI: confidence interval. RRD: rhegmatogenous retinal detachment. NA, not applicable; SF6, sulfur hexafluoride. ILM, internal limiting membrane. ^*∗*^Mann–Whitney test. ^*∗∗*^Kruskal–Wallis test.

## Data Availability

The data used to support the findings of this study are available from the corresponding author upon request.
